# Infection prevention practice in home healthcare: a mixed-method study in two Swiss home healthcare organisations

**DOI:** 10.1186/s12913-024-11111-y

**Published:** 2024-05-22

**Authors:** Lisa Brockhaus, Claudia Lötscher, Niklaus Daniel Labhardt

**Affiliations:** 1 Division of Clinical Epidemiology, Department of Clinical Research, University Hospital Basel, Basel, Switzerland; 2 Spitex Basel, Basel-Stadt, Switzerland; 3 Division of Infectious Diseases and Hospital Epidemiology, University Hospital Basel, Basel, Switzerland

**Keywords:** Infection prevention, Infection prevention and control, Home care, Home healthcare, Hand hygiene, Multi-drug resistant organisms

## Abstract

**Background:**

Infection prevention and control (IPC) research has long neglected the home healthcare sector with its unique challenges. This study aimed to gain an understanding of the barriers to the implementation of infection prevention practices relevant to this setting, the related attitudes, perceived relevance and priorities from the home healthcare worker perspective in Switzerland.

**Methods:**

The mixed-method study involved semi-structured interviews (*n* = 18) and an anonymous web-based survey (*n* = 144) among nursing assistants and nurses from two home healthcare organizations in northwest Switzerland. Questions in both sub-studies focused on perceived challenges to infection prevention practices, perceived relevance, and related attitudes and mitigation strategies. Using an exploratory-sequential design, survey questions were designed to quantify and complement the findings from the interview study.

**Results:**

Healthcare workers in these two organisations felt adequately protected, trained and supported by their organisations regarding IPC (survey agreement rates > 90%). General challenges to IPC in the home environment most agreed on were lack of cleanliness, lack of space, and the priorities of the patient to be respected (survey agreement rates 85.4%, 77.1%, and 70.8%, respectively). Practices and perceived challenges in the case of colonisation with multi-drug resistant organisms (MDRO) and potentially infectious diarrheal or respiratory illnesses varied highly regarding information transfer, use of protective equipment, and use and disinfection practices of multi-use equipment. Challenges to hand hygiene, sharps safety, waste management and decontamination of equipment did not feature as a prominent concern.

**Conclusions:**

This study is the first to characterise the implementation of infection prevention practices and the related challenges in home healthcare in Switzerland. Home healthcare workers describe various challenges related to infection prevention practices as largely manageable in their work routine, and generally show satisfaction with the support provided by their organisations regarding IPC precautions. Key findings regarding challenges amenable to interventions include uncertainty and inconsistency regarding the management of MDRO colonisation and acute illnesses, and gaps in information transfer. Those challenges may benefit from both organisational interventions and further research into the level of precautions that are appropriate to the home healthcare setting.

**Supplementary Information:**

The online version contains supplementary material available at 10.1186/s12913-024-11111-y.

## Background

The home healthcare (HHC) sector is rapidly expanding in many high-income countries, reflecting the demographic changes with an ageing population, as well as shifts from institutional to home-based care [1–3]. HHC is one of the fastest-growing segments of the Swiss healthcare sector. Over the last decade, the number of individuals receiving HHC has increased by more than 50% [4, 5]. Furthermore, due to structural and financial pressures on acute care, patients are being discharged from hospitals earlier, resulting in an increase in the complexity and severity of home care patients [6].

Infection prevention and control (IPC) guidance specific to the HHC setting is largely lacking [7–9]. Understanding the factors that limit the implementation of IPC practices in the HHC context is essential to adapt established infection prevention strategies to this unique setting, and to develop tailored interventions to address adherence where necessary. However, IPC implementation research has long neglected the heterogeneous HHC sector [8]. The limited research has largely focused on injection safety, while other precautions remain poorly studied [10]. The lack of IPC strategies tailored to the HHC setting is demonstrated by low adherence to established infection prevention guidelines developed in hospital contexts, such as to prevention bundles for central line-associated bloodstream infections, or hand hygiene standards [11–13]. Also, the lack of context-specific guidance was perceived as a major challenge by HHC organisations during the COVID-19 pandemic [14, 15].

In Switzerland, the HHC sector lacks structured IPC research and implementation efforts, with local organisations being responsible for the development of their individual IPC guidelines.

This study aimed to gain an understanding of the challenges to the implementation of IPC practices relevant to this setting, and the related attitudes, from the home healthcare worker (HHCW) perspective in Switzerland.

## Methods

The objectives of the study were to (i) explore the HHCWs’ perspectives on barriers to the implementation of various IPC practices, and their attitudes towards implementation including mitigation strategies, perceived relevance of obstacles, and priorities; and (ii) to quantify relevant key findings.

We conducted a mixed-method study using an exploratory-sequential design [16]. Approval was obtained from the University Basel Ethics Committee (2023 − 129).

Participants were recruited from two regional HHC organisations in the northwest of Switzerland, one providing services in a suburban area (organisation A) and one in an urban area (organisation B), with 177 and 611 employees, respectively. Both organisations largely provide general HHC services for adults, and serve areas with diverse socio-economic contexts. Both are privately run not-for-profit organisations with a governmental mandate.

Participation was limited to employees providing healthcare services, including nurses and nursing assistants with medical treatment competencies, such as wound care and blood glucose management, and nursing assistants without medical treatment competencies providing basic healthcare such as assisting with personal hygiene. Employees exclusively providing household help were excluded.

### Interview study

Interview participants were purposively sampled across the range of job profiles and training levels as outlined above. We aimed for a sample of 15–20 participants to reach code saturation [17], and finally stopped inclusion at 18 participants. No direct incentives were provided to the participants.

The interview guide was informed by our previous literature review [10], a field visit of the first author (LB), input from one HHC organisation (CL), and IPC expertise of the authors (LB, NDL). It was piloted on two HHCWs with subsequent refinements, and additional input from a researcher with vast experience in qualitative research was sought (JB).

The semi-structured interview started with an open question aiming to explore the participants’ personal definition of IPC precautions, and any aspects they spontaneously considered problematic. Then, 5–7 (depending on the participants’ job profile) questions about individual IPC practices and overarching issues were discussed focussing on aspects unique to the HHC setting. Two of them were phrased in the form of case vignettes ensuring practical understanding. Probing questions were asked to get more thorough descriptions of participants’ practices, and elicit a deeper understanding of their reasoning. Organisational and policy aspects were touched upon if perceived relevant by the participants, but were not the focus of the study. The interview concluded with open questions about any further aspects they considered important, and areas of improvement. The full interview guide is available in supplemental file 1.

The interviews were conducted by the first author in July 2023, in Swiss German, audio-recorded, and manually transcribed into Standard German. The analysis was performed using MS Excel.

Within the dataset, the analysis focussed on describing factors impacting IPC practices from the HHCW perspective, while also contextualising those with the participants’ reasoning and general attitudes, such as views on relevance and priorities.

Identification and analysis of themes were performed as outlined by Braun et al. using a 6-step thematic analysis [18]. Both an inductive (not driven by an interest in a specific IP practice) and a deductive (by IP practice) approach were used in the coding process and identification of themes. Themes were identified at the semantic level. Coding was performed and themes were discussed and agreed on by two study team members (LB, CL). To maximise validity, we used negative case analysis, synthesised member checking [18] with two participants, and triangulation with the survey data.

### Survey

17 survey items were designed to quantify the main themes identified from the interview study. Answering options were a 4-step Likert scale rating from ‘agree’ to ‘disagree’, or frequency of occurrence of specific situations, as appropriate. Questions were kept in jargon-free language and were piloted on 4 participants to ensure clarity.

The survey was delivered between 24 August and 21 October 2023 as a self-administered, anonymous, web-based online survey. All employees of the two organisations with the abovementioned job profiles were invited to participate, excluding nurses working exclusively in management roles. Eligible employees were invited to participate via e-mail by the HHC organisation management (directly in organisation A, and via team leaders in organisation B) including participant information. No incentives were paid to the participants, but they were provided time to complete the survey during working hours.

All survey results are reported descriptively using proportions. In the text, we report cumulative proportions of “agreeing” or “rather agreeing” to the respective item.

Furthermore, we performed a stratified analysis of all results by training level and by organisation. Therefore, responses were dichotomised to “agree/rather agree” versus “disagree/rather disagree”, and training levels were dichotomised to the two groups “participants without medical treatment competencies“ versus “participants with medical treatment competencies”. Results were analysed for significant differences using X2-test for trend, or Fisher’s exact test, as appropriate, and are only reported where significant differences (p < 0.05) were found.

Additional details on the survey methodology are provided in supplemental file 2 according to the CHERRIES-checklist [19].

## Results

Participant characteristics of the interview study (*n* = 18) and the survey (*n* = 144) are given in Table 1.

Among the employees contacted for the interview study, one declined to participate. Among the 10 licensed nurses included in the interview study, 5 had additional management roles. The mean duration of the interviews was 16 min (SD 5.4, range 6–30).

The survey was completed by 144 HHCWs, equivalent to an overall response rate of 24.4% of the eligible employees (53.8% in organisation A, and 18.6% in organisation B). The complete survey results are provided in the supplemental file 3.

**Table 1:** Participant characteristics of the interview study and survey

	Interview study (*n* = 18)	Survey (*n* = 144)
Age (mean)	47.8y (SD 12.0, range 27–63)	47.1y (SD 11.8, range 18–68) *(NA = 10*)
Sex: female (%)	17 (94.4%)	120 (85.1%) *(NA = 3)*
Organisation A (%)	8 (44.4%)	42 (30.6%) *(NA = 7)*
Years of experience in home healthcare (mean)	11.4y (SD 10.8, range 1–33)	10.2y (SD 8.8, range 0–41) *(NA = 15)*
Training level:		*(NA = 3)*
- Nursing assistants without treatment competencies (%)	4 (22.2%)	25 (17.7%)
- Nursing assistants with treatment competencies (%)	4 (22.2%)	36 (25.5%)
- Licensed nurses (%)	10 (55.6%)	80 (56.7%)

### General attitudes and perceptions

A key perception of participants was that implementation of infection prevention (IP) in terms of cleanliness or asepsis was not possible to the same extent as in the hospital setting. Participants then often went on to state their commitment to implementing precautions in the individual home, requiring their flexibility or even creativity:

*«I also work a lot with common sense, simply setting up the environment in a way that I can achieve the best possible results, knowing that this is not like in the textbook.»* (ID2)

However, some participants expressed a more dominant pragmatism resulting from the perceived limitations:

*«You just do it [hand hygiene] when you know it’s really necessary. Also because you know that you can’t do it quite as properly as in hospital.» (ID13)*

Participants also commonly expressed overall satisfaction with the precautions available for their protection, and IP implementation by the organisation. However, only few accounts did not include any aspect perceived as challenging IP practices in their setting.

95.8% of the survey participants agreed or rather agreed to feeling protected with the precautions in place in their organisation. 93.1% agreed or rather agreed to feeling safe to not transmit pathogens between patients with the precautions in place.

General challenges to IP practices eliciting the highest agreement in the survey were lack of cleanliness (85.4%), lack of space (77.1%), and the priorities of the patient having to be respected (70.8%). Participants with medical treatment competencies stated significantly more often being limited in their IP precautions by the space available in the home, compared to participants without medical treatment competencies (83.5% and 54.2%, respectively, *p* = 0.005).

### Relationship with the patient

Patient and HCW roles being different from the hospital setting was an overarching theme across conversations about specific IP precautions. Participants highlighted the greater self-determination of the patient in many care decisions, and the motivational work necessary if they felt changes were needed for IP reasons:

*«And also the understanding of the patient, you have to fight more for it. In the hospital you are the boss, at home the patient is the boss…» (*ID18)

However, there was broad consensus that in most cases, patients were very willing to comply if adequate explanations were given.

«*If you talk to them, there is always a solution. So I really try to adapt, so far it always worked out somehow.*» (ID6)

In the survey, 78.5% stated patients would usually be collaborative on IP precautions. 70.8% perceived the priorities of the patient having to be respected as a barrier to their IP management.

### Risk assessment and information transfer

Most participants stated they would treat all their patients the same with regard to IP practices. A frequent reasoning for this attitude was the perception that some diagnoses relevant to IPC might not be reported to them as HHCW in charge. An alternative view was vaguely being “more cautious” with severely immunocompromised patients.

Participants expressed diverse views on the reliability of information flow relevant to IPC for specific patients. In Switzerland, discharge letters and diagnoses lists are not routinely sent to HHC organisations by hospitals or general practitioners. Some felt confident that the relevant information would be available from these external documents, and transferred to their information system, while others saw a lack of relevant information:

*«So normally, when someone comes home from the hospital, the general practitioner, or relatives, actually contact the home care organisation…. in most situations this works well.»* (ID2)

*«Honestly, I haven’t heard of anyone leaving the hospital with MRSA [methicillin-resistant Staphylococcus aureus], and I can’t imagine that nobody has it. And we don’t realise it.»* (ID9)

Views on whether hospital discharge letters were easily available, or hard to obtain, were also divided, with occasional mention of the problem source being a hospital, GPs, or specific employees. It was also repeatedly pointed out that the legal basis for this information transfer seemed unclear:

*«That’s difficult - we don’t always get a diagnosis list from the hospital, it depends on who you communicate with, ‘oops, that’s data protection…’ »* (ID12)

Lack of medical information was perceived as a barrier to IP precautions by 55.6% of survey participants. When being asked about potential improvements, 78.5% and 85.4% saw a need for better information flow within the organisation, and between the organisation and other healthcare providers, respectively.

### Hand hygiene

Hand hygiene featured prominently as the most important IP precaution to participants. Implementation of hand hygiene was rarely an issue for participants. Key explanations provided for this perspective were good availability of hand sanitiser, either stored at the home or carried along, and a feeling of being appropriately trained on these precautions.

Participants often expressed confidence in their individual decisions on what level of hand disinfection was appropriate:

*«In any case, it can be implemented as I find it appropriate in home care… that the way I can disinfect my hands is sufficient.»* (ID2)

*«(…) because there are always situations where you have to do one more move, where it might make sense to disinfect your hands immediately afterwards, but then I’m just disinfecting my hands…sometimes that’s not feasible.»* (ID9)

While the risk of stains on furniture and floors with alcoholic hand sanitisers featured in some accounts, no other potential hassles were mentioned repeatedly. Time constraints were rarely mentioned.

Participants were divided on the indications and frequency of their hand washing in the patient’s home. Those who stated washing their hands occasionally provided individual strategies for drying their hands with something clean, including carrying along baby wipes, using toilet paper, or a cotton apron. Other participants described that they preferred disinfecting their hands or using gloves when they found the home too dirty to wash their hands, however acknowledging this was rarely the case.

*«We should [wash hands] before we prepare food…but with certain patients it’s pointless because it’s so dirty. So then I prefer to disinfect my hands. Or I put on gloves.»* (ID10)

While most participants described wearing gloves for limited indications such as intimate care or potential contact with body fluids, some also stated wearing gloves generously or even continuously for all care activities.

33.3% and 14.6% of survey participants stated using hand sanitisers more frequently, and less frequently, than recommended in the guidance, respectively. 57.6% used gloves more deliberately than as proposed in the guidance, and 21.3% stated using gloves for all care activities.

### Multi-drug resistant organisms

A case vignette of a patient with known Methicillin-resistant Staphylococcus aureus (MRSA) colonisation elicited diverse themes:

(1) The awareness that colonisation with multi-drug resistant organisms (MDROs) in their patients may be unreported, or undiagnosed.

(2) Focusing on good hand hygiene without applying any additional precautions, an approach for which some of the nurses with management roles also offered explanations for:

*«The patients take off their pants and reach into them… and then immediately touch other things again. In other words, we don’t actually know where anything has been distributed… And if we also don’t know that someone has MRSA or ESBL [Extended-spectrum beta-lactamase-producing Enterobacterales], and we only actually put the gloves on for personal hygiene, and otherwise, we touch things without gloves, then we can only hope that when we leave again and do our hand hygiene carefully, we don’t take anything with us. »* (ID9)

(3) A belief that guidelines for the management of specified MDRO were available at the organisation, defining in what situations additional precautions would be necessary.

(4) Uncertainty about the necessary precautions, and doubts about the relevance of the MDRO diagnosis depending on the care activity, were expressed by nurses with management roles:

*«There is a lack of clarity here. I do get this information when it is known at the patient’s discharge. They point out that the hygiene regulations are being strictly adhered to. But nothing special in any way… »* (ID17)

*«But we still have to look into it, and first have to read and think about where the bug is sitting and whether it is relevant for us… » (*ID8)

(5) A frequent view among health assistants was that they had not come across a MDRO diagnosis up to now.

In the survey, an overall 87.5% of participants believed that MDRO guidelines existed in their organisation. 66.7% of the participants stated having seen patients with known MDRO colonisation. This proportion was significantly higher in participants with medical treatment competencies (79.5%) than in those without (27.3%) (*p* < 0.001). Participants from organisation B also stated significantly more often having seen patients with known MDRO colonisation (*p* = 0.02).

### Potentially infectious acute illnesses

A case vignette of a patient with the notion of acute diarrhoea revealed two themes about IPC management of acute, potentially infectious illnesses:

(1) A key perception was that they had to make a new situation analysis when they visited the patients’ home:

*«…you can’t judge the situation just at once. You come in and pay attention, prepare yourself, so I read through everything, meaning then I might need extra gloves, and also make sure the sanitiser bottle is full, like that. You don’t do anything special.» (*ID16)

(2) In addition, most participants described carrying an “emergency kit” with personal protective equipment, provided by the organisation, that allowed them to react to unexpected situations.

Paying extra attention to hand disinfection was largely agreed on in the survey for diarrheal and respiratory acute illnesses (91.0% and 77.1%, respectively). Paying extra attention to disinfecting equipment was stated by 77.1 and 61.8%, respectively.

### Sharp safety

Participants stated unanimously that sharps disposal was generally well organised, with agency-provided sharp containers. Some spontaneously acknowledged that, similarly to the hospital setting, a residual risk of sharp injuries would always be present.

The rare cases in which issues arose showed three facets: (1), patients unable to comply with agreements, (2), caring for patients with intravenous drug use, and (3) plastic bottles being used if in the rare case, a container was not in place. However, participants readily presented their strategies to deal with those situations:

*«So we had one [person with substance use] who had all the mess in his room, and then we said he had to come into the kitchen for wound care, and then you’re safe.» (ID10)*

*«If we have patients where we know it won’t work, then we lock it away so that they can’t get to it.»* (ID13)

It was further emphasised that patients who self-managed blood glucose measurements and insulin injections were also responsible for proper disposal of their equipment, although HHC staff could advise them and organise proper disposal boxes. The financing of safety devices and equipment was occasionally brought up but was generally perceived as a manageable challenge. 92.4% of survey participants stated that sharps were usually safely disposed of in the patients’ homes.

### Waste management

Usually, waste disposal did not pose challenges to participants. They explained how materials contaminated with bodily fluids would be closed in a separate waste bag, and be disposed of in the household waste. Issues linked to this practice remained anecdotical across the accounts:

*«We also have patients who empty the syringe container into the bin and then put it back… » (ID10)*

In the survey, 83.2% stated that contaminated waste was usually being correctly disposed in the patients’ homes.

### Decontamination/reprocessing

When asked about the handling of multi-use equipment, the dominant theme was a preventive effort of not laying down equipment in the patient’s home, in general, or at least not in a place they considered dirty. This often required creativity, as the following quote illustrates:

*«There’s a loop at the back of the nursing bag where I hang the helmet and then I can put the tablet in there, so I don’t have to put it on the table.»* (ID6*)*

Multi-use equipment that was listed by participants included blood pressure meter, blood glucose meter, nurse bag and tablet. Participants described they would usually clean these items at the end of their working day, while cleaning strategies during the day were described inconsistently. However, this was rarely considered problematic:

*«I take it inside [into the patients’ home] and clean it in the evening…There might be something on it. I put it down and then pick it up again, well my hands are then disinfected, and then I put it in the car and take it to the next patient and put it down again… » (*ID11)

*«And you go in with everything, including your bag, which you put or hang somewhere, and then you take it back into the car, so certain things you just drag around.»* (ID13)

However, when survey participants were asked if they paid extra attention to disinfecting multi-use equipment after use in a patient colonised with MDRO, a patient with acute diarrhoea, or with acute respiratory symptoms, these statements were agreed to by 83.2%, 77.1%, and 61.8% of participants, respectively.

### Wound care and asepsis

Establishing a clean surface for equipment and materials was the dominant theme in wound care conversations. Challenges most frequently described were a general lack of space, often associated with a “messy household”, or a generally dirty household, while descriptions of pets, pet hair, open windows, or lack of light were occasional accounts. However, participants acknowledged that those situations were not the common case and solutions would normally be found:

*«There are the tidier and the less tidy households. But as a rule, I haven’t heard that this is a problem. I’ve also experienced a messy household, but that’s special, and even then I was able to make room for the dressings and drawing blood and so on.» (*ID17)

Participants emphasised adaptability and creativity were necessary and presented various strategies to create a satisfactory environment, including using newspaper piles, clean waste bags or packaging materials.

A second theme was the efforts to ensure the use of clean and appropriate wound care materials. Participants emphasised that some materials as well as equipment, such as single-use tweezers, were not paid for by health insurance. Re-sealing of opened materials to reduce costs was commonly reported in this context. Participants also mentioned organising a clean box from their HHC organisation to store all wound care materials and equipment in the home.

In the survey, the three most frequent barriers to clean wound care (rated “frequent” or “sometimes”) were lack of space to make a clean surface (68.7%), positioning of the patient difficult (63.5%), and lack of cleanliness in the home (59.2%).

### Contextual themes: knowledge level, adherence, and COVID-19-related challenges

IP-related challenges during the COVID-19 pandemic were not explored explicitly and did not feature prominently in the accounts. When it was occasionally brought up spontaneously, the most common theme was not knowing whether a patient could be sick due to COVID-19 and infectious, and not being able to do anything about this:

*«And he coughs right in my face (…)- then I’ve already told customers to test, and they didn’t know how to test, let alone had relatives to bring them to the test centre or anything else…and then we just let it be.»* (ID18)

Participants unanimously rated their knowledge level about hygiene practices as sufficient or good, a perception that was shared by nurses with management roles who were asked about the knowledge level of their team. Justifications provided most frequently included training sessions, work supervision, the existence of guidance and knowing where to look up questions, and a positive feedback culture in the team.

Although not explicitly asked, an attitude of perceived importance of hygiene precautions and adherence was visible in many accounts.

*«…you usually only find out afterwards whether there is a germ or something, … so I am very rigorous and strict, I don’t want to bring anything and take anything with me.» (ID6)*

This view was echoed by nurses with management roles:

*«Looking back on the corona period, it took a very long time for our people to become infected at all, and that was only when the measures were relaxed and people were able to meet again in their private lives… That’s why I believe that a minimum of precautions is being observed very carefully.» (ID9)*

In the survey, 97.2% of participants perceived their own knowledge level to be sufficient for their work routine.

## Discussion

### Summary of key findings

This study characterises IP implementation in the Swiss HHC setting with the following key findings: (i) HHCW in these two organisations felt sufficiently protected, trained and supported by their organisations regarding IPC, and felt committed to adapting their precautions to the diverse conditions they encounter in their work; (ii) Challenges of hand hygiene, sharps safety, waste management and decontamination of equipment did not feature prominently as a HHCW concern; (iii) Practices, perceived relevance and challenges in the case of colonisation with MDRO or potentially infectious diarrheal or respiratory illnesses were highly varied regarding information transfer, use of protective equipment, and use and disinfection practices of multi-use equipment; and (iv) main perceived challenges to IP practices in general in the home environment were lack of cleanliness, lack of space, and the priorities of the patient to be respected.

### Strengths and limitations

Our study design allowed us to not only describe IP practices and self-perceived barriers in this setting, but also to provide a nuanced picture with regard to relevance and priorities as perceived by the HHCWs which we identify as a strength of our study.

The representativeness of this study may be limited due to the relatively small number of participants, the inclusion of only two organisations, and the rather low survey response rate in organisation B. However, we included two organisations characteristic for the Swiss setting: They are privately run not-for profit organisations with a governmental mandate, are members of the national umbrella association of HHC organisations, represent a typical range of organisation size in the Swiss setting, and serve socioeconomically diverse areas. The two organisations differ regarding area served (urban versus suburban), size (611 versus 177 employees), and, importantly, internal management mechanisms. The latter may explain the lower survey response rate in organisation B, with survey invitations being distributed indirectly via team leaders, in contrast to direct distribution by the management in organisation A. With the exception of the proportion of employees stating seeing patients with MDRO colonisation, we found no significant differences in the survey responses when stratifying for organisation. This finding makes us confident that the key findings may be transferable to other HHC organisations at least within the Swiss context.

Furthermore, it is possible that some potential gaps in a rather favourable picture were not disclosed. Particularly, we found a discrepancy in self-declaration of decontamination practices of equipment when seeing patients with MDRO or acute illnesses between the interview study and the survey results that may be explained by some desirability bias. However, in all other practices under question, survey results aligned with the qualitative findings.

Lastly, the study deliberately focused on the HHCWs perspectives, meaning that factors that currently may lack awareness in the setting are potentially not identified as barriers by participants.

### Comparison with existing literature

Key challenges highlighted by participants in this study have been described previously in other high-income countries, most notably including space and cleanliness in the home [20–23], patient priorities [21, 24], and inconsistency regarding management of MDRO colonisation [21, 24]. When compared to this limited evidence from other high-income countries, it is noticeable that participants in our study largely describe the challenges encountered as manageable rather than overwhelming. For example, survey studies from the US reported multiple barriers to IPC practices upon each home visit [20, 22]. Apart from inter-country differences, this potential discrepancy may be explained by our study design putting encountered barriers into perspective by asking interview participants to elaborate on the perceived relevance of factors they identified, and by differences in how the survey questions were phrased. Furthermore, our study does not identify sharps safety as a predominant IP concern for HHCW in this setting, a finding that contrasts with the focus of previous research [10]. We argue that with device improvements and improved knowledge about transmission of blood-borne pathogens over the last decades, other concerns have become more dominant.

Literature about IP management of patients with MDRO colonisation in HHC is particularly scarce. One study from the US revealed that practices of taking equipment into the home or using dedicated equipment varied widely [25], a finding that aligns with our study. Another study from the US showed 48% of HHC nursing bags were contaminated with bacterial pathogens on the inside, of which 6% were MDRO [26]. To our knowledge, effectiveness of the decontamination practices for equipment in HHC has not been studied. Inconsistency around the management of various MDROs reflects evidence gaps that are not limited to the HHC setting: the use of contact precautions is increasingly questioned as evidence for their effectiveness is missing even in the hospital setting [27–29].

Challenges related to IP practices during the Covid-19 pandemic, somehow surprisingly, did not spontaneously dominate participants’ accounts. However, our study focused on practices rather than the policy and organisational level, and thus does not conflict with findings of other publications describing the policy response in and for HHC during the Covid-19 pandemic as inappropriate [14, 15].

### Implications for future research, and policy and practice

Identified barriers around information transfer, and inconsistency regarding the management of MDRO or acute illnesses, may primarily benefit from operational interventions. This may include clarification of the legal situation for health records transfer to HHC organisations, at least in the Swiss context. From a technical perspective however, evidence about what level of precautions is effective in and appropriate to the HHC setting is further missing. This is particularly true for the management of various MDROs as an increasingly relevant challenge to all healthcare sectors [9]. Robust epidemiological and clinical data on colonisation and healthcare-associated infections in the setting are missing [7, 30, 31], and would provide a very first step towards clarification of precautions appropriate in this context.

## Conclusion

This study is the first to characterise the implementation of IP practices and the related challenges in HHC in Switzerland. HHCW describe various specific challenges related to IP practices as largely manageable in their work routine, and generally show satisfaction with the support provided by their organisations regarding IPC precautions. Key findings regarding challenges include uncertainty and inconsistency around the IP management of MDRO colonisation and acute illnesses, and gaps in information transfer. Those challenges may benefit from both organisational interventions and further research into the level of precautions that are appropriate to the HHC setting.

### Electronic supplementary material

Below is the link to the electronic supplementary material.

Supplementary Material 1

Supplementary Material 2

Supplementary Material 3

## Data Availability

The original data of this study are available from the correspondig author on reasonable request.

## Abbreviations

HHC: Home healthcare
HHCW: Home healthcare worker
IP: Infection prevention
IPC: Infection prevention and control
MDRO: Multi-drug resistant organisms
MRSA: Methicillin-resistant Staphylococcus aureus
