# Supplementary material for: Infection prevention practice in home healthcare: a mixed-method study in two Swiss home healthcare organisations
Source: BMC Health Serv Res. 2024 May 22;24:657. doi: 10.1186/s12913-024-11111-y (PMC11112953; doi:10.1186/s12913-024-11111-y)
Supplement: Supplementary file 1 — Supplementary Material 1 [file 12913_2024_11111_MOESM1_ESM.docx]

**Interview guide (translation from German to English)**

Opening question:

What does “infection prevention” or “hygiene precautions” mean in your work routine?

Optional probes:

1. What other issues around hygiene precautions are also important?
2. How important do you feel is that in your work routine?
3. How do you decide whether this is important in a specific patient?

Hand hygiene:

How do you use hand hygiene in your routine work? What is particularly important to you?

Optional probes:

1. Can you give an example?
2. What is stopping you from doing this?
3. Are there situations you want to wash your hands?

Personal protective equipment:

Vignette: A patient has acute diarrhea since yesterday. What does that mean to you regarding hygiene precautions?

Optional probes:

1. How can you deal with this?
2. How do you communicate this to the patient?
3. Can you give an example?

Disinfection/Reprocessing:

Which equipment or materials do you use multiple times during the day?

Optional probes:

1. What do you then do with these materials?
2. What options do you have?
3. What do you do with your bag? With your tablet?

Multidrug-resistant organisms

Vignette: A patient is discharged from hospital and has been diagnosed with an MRSA (a resistant hospital germ). What does this mean for your work routine?

Optional probes:

1. How do you deal with that, then?
2. How do you communicate this to the patient?
3. Can you give an example?

Waste management

How do you dispose of materials contaminated with bodily fluids, e.g. wound dressings or indwelling catheter bags?

Optional probes:

1. How do you deal with this, then?
2. What is the role of the patient?
3. Can you give an example?

Injection safety (only nurses / nursing assistants with treatment competencies)

How do you deal with needles and sharps, e.g. for blood glucose monitoring?

Optional probing:

1. How do you dispose of this, then?
2. What is the role of the patient?
3. How do you recognise whether this home is at risk for unsafely disposed needles?

Asepsis (only nurses / nursing assistants with treatment competencies)

How do you deal with hygiene precautions when doing a dressing in the patients’ home?

1. How do you deal with this, then?
2. Can you give an example?
3. How do you decie whether that’s important in the situation?

Knowledge level:

How would you rate your personal knowledge level about hygiene precautions?

Probing:

1. Where do you get this information from?
2. Is this information sufficient for your needs?
3. Can you apply this knowledge in your work routine?

Further aspects

Is there any other important issue around hygiene precautions we have not talked about yet? Something you want to add?

Improvement suggestions

Is there anything you would like to see being improved for your work routine, regarding hygiene precautions?
