# Supplementary material for: Infection prevention practice in home healthcare: a mixed-method study in two Swiss home healthcare organisations
Source: BMC Health Serv Res. 2024 May 22;24:657. doi: 10.1186/s12913-024-11111-y (PMC11112953; doi:10.1186/s12913-024-11111-y)
Supplement: Supplementary file 2 — Supplementary Material 2 [file 12913_2024_11111_MOESM2_ESM.docx]

**CHERRIES (Checklist for Reporting Results of Internet E-surveys) – Checklist**^(1)^

|  | **Checklist item** | **Reported on Page no.** | **Additional comments** |
| --- | --- | --- | --- |
| **Design** |  |  |  |
| IRB approval and informed consent process | IRB approval | 2 |  |
|  | Informed consent |  | Participant received a study information pdf file explaining the purpose and process of the study. Informed consent was then obtained at the beginning of the survey, using a stop button function in the case of disconsent. |
|  | Data protection | 3 | A generic survey link was used |
| Development and pre-testing | Development and testing | 3 |  |
| Recruitment process and description of the sample having access to the questionnaire | Open vs. closed survey |  | Open survey |
|  | Contact mode |  | The survey link and participant information and survey link was sent out via E-mail, by the management of both organisations, indicating that they do so in collaboration with the external study team. |
|  | Advertising the survey |  | The survey was advertised by the management of both organisations, indicating that the survey was voluntary but participation encouraged and supported by the management. |
| Survey administration | Web/E-mail | 3 |  |
|  | Context |  | The survey was posted using the REDcap survey tool. |
|  | Mandatory/voluntary | 3 | voluntary |
|  | Incentives | 3 |  |
|  | Time/date | 3 |  |
|  | Randomisation of items or questionnaires |  | No randomized or alternating questions |
|  | Adaptive questioning |  | Yes, depending on job profile, and personal experience with MDRO, selected questions were omitted |
|  | Number of items | 3 |  |
|  | Number of screens (pages) |  | Variable between topics, mean 4 items (range 1-9) per page |
|  | Completeness check |  | No items were mandatory except for the consent button. Only records with complete submission, i.e. using a “survey complete” button at the end of the survey, were considered. Non-responses to specific items were labeled NA. |
|  | Review step |  | A “back” button was available to participants. No summary of answers was displayed to participants. |
| Response rates | Unique site visitor |  | An IP check was not possible using a generic survey link. With the consent button, participants confirmed not having taken part in the survey before. |
|  | View rate |  | Not possible (first survey page visitors not identifiable) |
|  | Participation rate |  | Not possible (first survey page visitors not identifiable). |
|  | Completion rate | 3 | Response rate of users who finished the survey / number of invitations is reported |
| Preventin multiple entries from the same individual | Cookies used |  | no |
|  | IP check |  | No. With the consent button, participants confirmed not having taken part in the survey before. |
|  | Log file analysis |  | No |
|  | Registration | N/A |  |
| Analysis | Handling of incomplete questionnaires |  | Only completed questionnaires were analysed. Questionnaires which terminated early without ticking the “complete” button were not analysed. |
|  | Questionnaires submitted with an atypical timestamp | N/A |  |
|  | Statistical correction |  | No. |
|  |  |  |  |
|  |  |  |  |
|  |  |  |  |

1. Eysenbach G. Improving the quality of web surveys: The Checklist for Reporting Results of Internet E-Surveys (CHERRIES). J Med Internet Res. 2004;6(3):1–6.
