# Supplementary material for: Infection prevention practice in home healthcare: a mixed-method study in two Swiss home healthcare organisations
Source: BMC Health Serv Res. 2024 May 22;24:657. doi: 10.1186/s12913-024-11111-y (PMC11112953; doi:10.1186/s12913-024-11111-y)
Supplement: Supplementary file 3 — Supplementary Material 3 [file 12913_2024_11111_MOESM3_ESM.docx]

Supplemental file 3. Full survey results

| **Survey item** |  |
| --- | --- |
|  | **% agree** l **% rather agree** |
| 1. I feel protected with the hygiene precautions in place at the organisation | 63.9 l 31.9 |
| 2. I feel safe not to transmit pathogens between patients with the precautions in place at the organisation | 52.1 l 41.0 |
| 3. I having sufficient knowledge of hygiene precautions for my work routine | 74.3 l 22.9 |
|  |  |
|  | **% agree** l **% rather agree** |
| 4. Barriers impacting hygiene precautions in my work routine: |  |
| - Lack of space | 26.4 l 50.7 |
| - Lack of cleanliness | 39.6 l 45.8 |
| - Priorities of the patient to be respected | 22.9 l 47.9 |
| - Priorities of the relatives to be respected | 13.2 l 46.5 |
| - Lack of time | 19.4 l 42.4 |
| - Lack of medical information | 16.7 l 38.9 |
| - Lack of materials and equipment needed at the visit | 18.1 l 36.9 |
|  |  |
|  | **% agree** |
| 5. I use hand sanitisers |  |
| - In more situations than in the guidance | 33.3 |
| - Exactly in the situations defined in the guidance | 50.0 |
| - In less situations than in the guidance | 14.6 |
| 6. I use gloves: |  |
| - Only in limited, specified situations | 19.4 |
| - More deliberately depending on care activity, patient, and environment | 57.6 |
| - For all care activities | 21.3 |
|  |  |
|  | **% agree** l **% rather agree** |
| 7. Sharps are usually correctly disposed in the patient’s home | 66.7 l 25.7 |
| 8. Contaminated waste is usually correctly disposed in the patient’s home | 36.1 l 47.2 |
| 9. Patients are usually willing to collaborate on hygiene issues | 20.8 l 57.7 |
|  |  |
|  | **% yes** |
| 10. I have had patients with known MDRO colonisation | 66.7 |
|  |  |
|  | **% agree** l **% rather agree** |
| 11. MDRO guidelines exist at my organisation | 44.4 l 43.1 |
| 12. MDROs are not problematic in home healthcare | 9.7 l 37.5 |
|  |  |
|  | **% yes** l **% case-by-case** |
| 13. Precautions I take with a patient known for MDRO colonisation: |  |
| - I pay extra attention to hand disinfection | 92.7 l 4.2 |
| - I take only the minimal equipment necessary into the home | 68.4 l 24.2 |
| - I pay extra attention to disinfecting equipment | 83.2 l 11.6 |
| - I use extra protective equipment (e.g. gloves, aprons) | 84.4 l 13.5 |
|  |  |
|  | **% yes** l **% case-by-case** |
| 14. Precautions I take with a patient with acute diarrhoea: |  |
| - I pay extra attention to hand disinfection | 91.0 l 3.5 |
| - I take only the minimal equipment necessary into the home | 55.6 l 28.5 |
| - I pay extra attention to disinfecting equipment | 77.1 l 12.5 |
| - I use extra protective equipment (e.g. gloves, aprons) | 62.5 l 31.9 |
|  |  |
|  | **% yes** l **% case-by-case** |
| 15. Precautions I take with a patient with upper respiratory infection symptoms: |  |
| - I pay extra attention to hand disinfection | 77.1 l 11.8 |
| - I take only the minimal equipment necessary into the home | 34.0 l 34.7 |
| - I pay extra attention to disinfecting equipment | 61.8 l 25.7 |
| - I wear a mask | 36.8 l 52.8 |
| - I ask the client to wear a mask | 31.9 l 55.6 |
|  |  |
|  | **% frequently (>30%)** l **sometimes (10-30%)** |
| 16. Barriers impacting clean wound care during the last month of my work: |  |
| - Lack of space to make a clean workspace | 20.0 l 48.7 |
| - Lack of cleanliness in the home | 12.2 l 47.0 |
| - Positioning of the patient difficult | 22.6 l 40.9 |
| - Lack of the necessary materials or equipment | 7.9 l 28.1 |
| - Materials not stored in clean conditions | 5.3 l 22.1 |
| - Patient not willing to collaborate on hygiene practices | 3.5 l 23.9 |
| - Pets potentially interfering with care | 2.6 l 13.9 |
| - Scheduled time insufficient | 3.5 l 15.8 |
|  |  |
|  | **%agree** l **% maybe** |
| 17. Improvement suggestions |  |
| - More frequent coaching at work | 11.1 l 36.8 |
| - More frequent training/educational activities | 24.3 l 51.4 |
| - Better communication on precautions between HHC organisation and other providers | 45.1 l 40.3 |
| - Better communication on precautions within HHC organisation | 41.7 l 36.8 |
| - Better guidance on precautions in specific situations | 41.0 l 26.4 |
| - Better availability of workwear, including trousers | 43.1 l 28.5 |
| - IPC expert /contact person within the organisation | 38.2 l 37.5 |

HHC: home health care; IPC: infection prevention and control; MDRO: multi-drug resistant organisms

Respondent numbers are n= 144 except for item 13 (n = 96) and item 16 (n = 116)
